# Suprasellar Mature Cystic Teratoma Mimicking Rathke’s Cleft Cyst: A Case Report and Systematic Review of the Literature

**DOI:** 10.3389/fendo.2021.731088

**Published:** 2021-09-30

**Authors:** Shenzhong Jiang, Zhaojian Wang, Yan You, Renzhi Wang, Xinjie Bao

**Affiliations:** ^1^ Department of Neurosurgery, Peking Union Medical College Hospital, Chinese Academy of Medical Sciences and Peking Union Medical College, Beijing, China; ^2^ Department of Pathology, Peking Union Medical College Hospital, Chinese Academy of Medical Sciences and Peking Union Medical College, Beijing, China

**Keywords:** mature cystic teratomas, sellar region, rare lesion, neuropathology, case

## Abstract

In this article, we present a 31-year-old female who presented with intermittent headache and oligomenorrhea of over 10 years’ duration. Imaging revealed a large suprasellar mass with sellar extension. The patient underwent an endoscopic endonasal trans-sphenoidal surgery to resection of the mass. Clinical, radiological, and operative findings from this patient were initially considered to be Rathke’s cleft cyst (RCC). However, postoperative histological examinations revealed a mature cystic teratoma. No radiotherapy was performed after surgery. At the most recent follow-up, approximately 1 year later, the patient is doing well with no headache and no recurrence of the teratoma.

## Introduction

Teratomas are a type of germ cell tumor (GCT) differentiating from three germ layers. Central nervous system teratomas are very rare, accounting for 0.2%–0.9% of all intracranial tumors (1). According to *The 2016 WHO Classification of Tumors of the Central Nervous System*, teratomas can be classified into three types: mature, immature, and teratomas with malignant transformation (2). Mature teratomas are benign tumors that contain well-differentiated tissues from at least two germinal layers which can be divided into two subtypes: mature solid teratomas and mature cystic teratomas (MCT); the former is exceedingly rare. The latter accounts for about 0.04% to 0.7% of all intracranial tumors (3, 4). Mature teratoma recurrence rate is extremely low in cases of complete resection and usually occurs within 1 year after treatment (5), and the 10-year survival rate is 93% (6). Most of the intracranial MCTs have been found to occur in the midline structures, and the pineal area is the most frequent site (7, 8). Suprasellar MCTs have rarely been reported. Here, we describe an unusual case of a large suprasellar MCT mimicking Rathke’s cleft cyst, and conduct a systematic review of eight cases of MCTs in the sellar region (**Tables 2** **–** **4**). We hope to shed new light for physicians on the diagnosis and treatment of this rare disease.

## Case Presentation

### History and Examination

**Figure 1:**
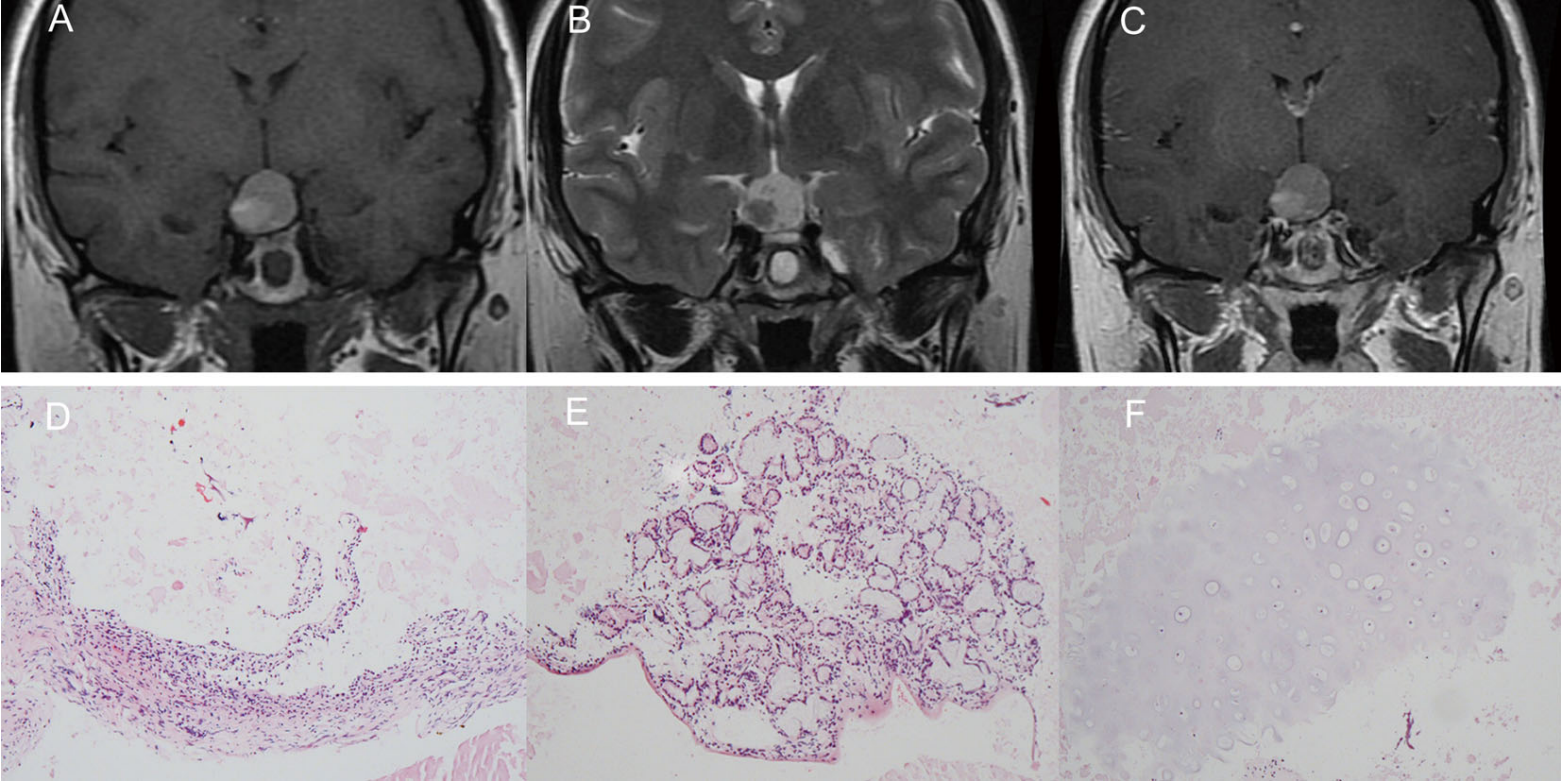
The lesion signal characteristics on magnetic resonance imaging were isointense on T1-weighted imaging and hyperintense on T2-weighted imaging. No obvious gadolinium enhancement was noted **(A–C)**. Pathological findings: on a background of abundant myxoid stroma, we can see fibrous cyst walls lined with simple cuboidal and short columnar epithelium (H&E ×100, **D**), a mass of mucous acinous cells **(E)**, and some chondroid tissue **(F)**.

A 31-year-old female was admitted to our hospital complaining of oligomenorrhea and increasing headaches. She reported an 11-year history of intermittent headache (visual analog scale, with 10 as the worst pain, of 4/10 points), which used to be precipitated by fatigue and were alleviated by rest or non-steroidal anti-inflammatory drugs (NSAIDs). When the headaches increased in frequency and intensity and were accompanied by mild nausea, culminating in a headache lasting for 1 week with no relief from NSAIDs, the patient sought medical attention. She denied vision loss, visual field defects, polyuria, lactation, central obesity, or acromegaly during the course. The general physical examination was completely normal, and the neurologic examination showed no focal signs. A brain magnetic resonance imaging (MRI) scan with contrast was performed, demonstrating a 19 mm × 24 mm × 23 mm irregular suprasellar lesion with slight intrasellar extension. The lesion signal characteristics were isointense on T1-weighted imaging and hyperintense on T2-weighted imaging. No obvious gadolinium enhancement was noted (**Figures 1A–C**). Endocrine workup showed that the levels of pituitary hormones were within normal limits (**Table 1**).

**Table 1:** Results of endocrine examinations before and after surgery.

Test	Reference range	Before surgery	3 months after surgery
		Value	Value
Sex hormone			
LH	1.20–103.03 IU/L	10.08	19.36
FSH	<30.34 IU/L	6.57	10.33
E2	27–433 pg/ml	60	130
P	0.38–29.26 ng/ml	0.21↓	1.73
PRL	<30 ng/ml	16.72	4.92
ACTH related			
ACTH	0–46 pg/ml	18.9	10.5
F	4.0–22.3 μg/dl	17.28	12.35
Thyroid function			
FT3	1.80–4.10 pg/ml	3.17	2.67
FT4	0.81–1.89 ng/dl	1.081	1.222
T3	0.66–1.92 ng/ml	1.179	0.824
T4	4.30–12.50 μg/dl	7.6	8.06
TSH	0.38–4.34 μIU/ml	3.154	2.335

LH, luteinizing hormone; FSH, follicle-stimulating hormone; E2, estradiol; P, progesterone; PRL, prolactin; β-HCG, β-human chorionic gonadotropin; ACTH, adrenocorticotropic hormone; F, cortisol; FT3, free triiodothyronine; FT4, free thyroxine; T3, triiodothyronine; T4, thyroxine; TSH, thyroid-stimulating hormone.

### Surgical Biopsy and Histological Findings

Endoscopic trans-sphenoidal surgery was performed. In the procedure, the cyst was observed to be predominantly suprasellar in location. It contained ivory-whitish viscous material and was resected. Hematoxylin–eosin staining is as follows: on a background of abundant myxoid stroma, we can see the following components: fibrous cyst walls lined with simple cuboidal and short columnar epithelium (H&E ×100, **Figure 1D**), a mass of mucous acinous cells (**Figure 1E**), and some chondroid tissue (**Figure 1F**).

### Postoperative Course

The postoperative course was uneventful, with the headaches completely resolving after surgery. During the 1-year follow-up, our patient is well and there is no evidence of recurrence.

## Discussion

In the case report, we present a unique and rare case of MCT mimicking Rathke’s cleft cyst (RCC) of the sellar region in terms of clinical manifestations and neuroimaging.

According to *The 2016 WHO Classification of Tumors of the Central Nervous System* (2), teratomas are a subset of intracranial germ cell tumors and rarely present as pure teratomas (rather than mixed germ cell tumors). Teratomas can be classified into three types: mature, immature, and teratomas with malignant transformation. Intracranial teratomas are rare space-occupying lesions that account for about 0.5% of all intracranial tumors. MCTs are a subset of these neoplasms, and their occurrence in the brain is even rarer. They are benign tumors that contain well-differentiated tissues from at least two germinal layers. MCTs occur more frequently during the first or second decade of life, and there is a clear male predominance (4:1). Most intracranial MCTs occur in midline structures, most frequently in the pineal region (7).

MRI is the first choice of neuroimaging in the diagnosis of RCC. On MRI, RCCs often appear as well-demarcated, centrally located spherical or ovoid lesions of the sellar region with nodules inside the cyst occasionally. The majority of these smooth contoured cysts are unilobar with a diameter ranging between 5 and 40 mm (mean approximately 17 mm) (9). MRI signal intensity varies and is highly dependent on the biochemical nature of intracystic contents, which can range from clear, CSF-like fluid to thick, mucoid material (10, 11).

In the present case, RCC was suspected prior to the histological examinations of the tumor because the gender, age, clinical presentations, and neuroimaging characteristics aligned with a diagnosis of RCC.

Suprasellar MCTs are relatively rare. MCTs occur more frequently during the first or second decade of life. Rarely, reported cases have occurred on the third or fourth decade of life (5). Overall, these tumors appear to be more common in men, with a finding of 79.7% in men versus 20.3% in women (6). Moreover, the tumor mimicking RCC is a further peculiarity of the case.

The published case reports and series written in English that focus on suprasellar MCTs are limited. Therefore, we performed a comprehensive literature review of related articles and identified eight patients with a diagnosis of MCT, summarizing the data of clinical manifestations (**Table 2**), pituitary function (**Table 3**), MRI signal features (**Table 3**), and treatment (**Table 4**) of this lesion.

**Table 2:** Demographic data and clinical presentation from published reports.

Patient	Author	Year	Country	Age/sex	Presentation	Other manifestation
1 (12)	Li et al.	2015	China	13/F	Polyuria, polydipsia, and amenorrhea	Headache, blurred vision, short stature
2 (8)	Sweiss et al.	2013	USA	57/M	Vision impairment	Left-sided facial weakness, ataxia, and short-term memory loss, seizure
3 (13)	Vendrell et al.	2010	France	18 months/M	Bilateral decreased visual acuity and hyperphagia	
4 (14)	Kim et al.	2010	Korea	17/M	Polyuria and polydipsia with severe thirst, headache, and diplopia	
5 (15)	Muzumdar et al.	2001	India	26/M	Headache, vision impairment	Short stature, weight gain
6 (16)	Araki et al.	2000	Japan	3 months/M	Fontanelle bossing	Accelerated deep tendon reflexes, incomplete head control
7 (17)	Narayanam et al.	2012	India	7/F	Seizures, precocious puberty, headache, and vomiting	Irritable
8 (18)	Tobo et al.	1981	Japan	14/M	Diabetes insipidus/panhypopituitarism	
9	Current case	2021	China	31/F	Headache, oligomenorrhea	

F, female; M, male.

**Table 3:** Pituitary function and pituitary magnetic resonance imaging data from published reports.

Patient	Pituitary function	Size	Location	T1	T2	T1 contrast	CT
1	T4↓, TSH↑, PRL↑	Large	Sellar and suprasellar	Cystic: hypointense	Heterogeneous hyperintense	Cystic: rim enhancement; solid: evidently enhanced	Hypodense
2	FSH↑, LH↑	Large	Intra- and suprasellar	NA	NA	NA	NA
3	PRL↑, TSH↑, T3↑	Large	Endosuprasellar	Mixed intensity	Mixed intensity	Intense enhancement	Hyperdense due to calcification
4	ADH↓	Large	Sellar and suprasellar	NA	NA	Partial enhancement	NA
5	Panhypopituitarism	Large	Suprasellar	Hyperintense	NA	NA	Hypodense with peripheral rim of calcification
6	Normal	Large	Suprasellar	Hypointense	NA	NA	NA
7	Normal	Large	Suprasellar	Isointense	Isointense	No enhancement	Hypodense
8	Panhypopituitarism	NA	Sellar and suprasellar region	NA	NA	NA	A mass in the suprasellar region with contrast enhancement
9	Normal	Large	Sellar and suprasellar	Iso/hyperintensity	Hyperintensity with a hypointense nodule	No enhancement	–

Size: large = large tumor size (>1 cm).

T3, triiodothyronine; T4, thyroxine; TSH, thyroid-stimulating hormone; LH, luteinizing hormone; FSH, follicle-stimulating hormone; PRL, prolactin; ADH, anti-diuretic hormone; NA, not available.

**Table 4:** Treatment and outcome of patients from published reports.

Patient	Surgery	Tumor contents	Pathology	Outcome	Complication
1	Right pterional approach	Dark yellow fluid; hair and whitish fat material	Mature teratoma	Total resection/vision improved	Transient DI
2	Right pterional craniotomy/trans-sylvian approach	Thick and yellow oil-like fluid, yellow clumps of hair embedded within fatty deposit	Mature cystic teratoma	Incomplete resection followed by external beam radiotherapy and stereotactic radiosurgery/significantly improved neurological status and vision	No
3	TSS	Teeth	Mature teratoma	Normal neurological examination except loss of visual acuity in the left eye	No
4	TSS		Mature teratoma	Followed by chemotherapy and radiotherapy	NA
5	Sublabial trans-sphenoidal approach	Fat, bony septation, keratinaceous flakes	Mature teratoma	Total resection/vision improved, normal visual field, headache gone	No
6	Surgery	NA	Mature teratoma	Total resection/panhypopituitarism and diabetes insipidus	Hydrocephalus/complete blindness
7	Left pterional approach	Whitish structure	Mature teratoma	Total resection/headache gone, seizure-free, regression of precocious puberty	No
8	Craniotomy	Bone, cartilage, and several hairs	Mature teratoma	NA	NA
9	TSS	Ivory-whitish viscous materials	Mature cystic teratoma	Total resection/headache resolved	No

TSS, trans-sphenoidal surgery; NA, not available.

The most prominent symptoms at diagnosis are neurological defects (six of eight patients), particularly visual disturbance (five of eight). Headache (three of eight) and diabetes insipidus (three of eight) were also commonly seen. One patient reported amenorrhea. Regarding MRI appearance, signal intensities on T1WI and T2WI vary from case to case. In some cases, inclusions like teeth, fat, and calcification can be detected (13, 15). Variable enhancement with contrast was reported in three patients. Liu et al. (19) suggest that mature teratoma on MRI is an ovoid or irregular mass with or without multilocularity and has mixed signals derived from different tissues. The presence of fatty tissue or multilocularity is a characteristic feature of teratoma. The tumor usually presents with heterogeneous hyperintensity on T1W images and non-enhanced or moderate enhanced multilocularity on T1W images with contrast. However, Chiloiro et al. (5) suggest that teratomas appear as low-intensity heterogeneous mass in T1- and T2-weighted magnetic resonance imaging, with variable enhancement after contrast administration. Surgery was performed in all patients, two of which were followed by radiotherapy or chemotherapy. Only one patient reported hydrocephalus and blindness during follow-up.

Neuroimaging characteristics of teratomas are not of high specificity, which make it difficult to distinguish mature teratomas from other intracranial neoplasms located in the suprasellar region that include other GCTs (germinoma, choriocarcinoma, embryonal carcinoma, and endodermal sinus tumor), craniopharyngioma, and RCC. Therefore, our case highlights the importance of obtaining a histological diagnosis to differentiate teratomas from other lesions.

Histologically, MCTs are commonly multicystic, contain sebaceous fluid, and are identified by the presence of differentiated ectodermal (skin, hair, brain), mesodermal (muscle, fat, teeth, bone, cartilage), and/or endodermal elements (mucinous and ciliated epithelium). All three layers may not be seen in every case of teratoma. The differential diagnosis includes dermoid cysts, epidermoid cysts, colloid cysts, immature teratomas, and teratomas with malignant transformation.

For this case, our preoperative diagnosis was Rathke’s cleft cyst, and given the absence of hair, skin, or teeth, the intraoperative findings seemed to confirm our primary diagnosis. However, MCTs were confirmed by the histological examination of the specimen when cyst walls lined with simple cuboidal and columnar epithelium, a mass of mucous acinous cells (salivary glands), and cartilage were identified. Our case highlights the importance of obtaining a histological diagnosis to differentiate MCTs from other lesions. It would also be important to exclude the presence of additional germ cell components, which would require additional treatment postresection.

The typical treatment for mature teratomas is neurosurgical excision because of their benign behavior (20), which was successfully done in this case. It is well advised to perform radical excision as the long-term outcome is excellent. Mature teratoma recurrence rate is extremely low in cases of complete resection and usually occurs within 1 year after treatment (5). Sano (6) reported that the 10-year survival rate for mature teratomas is 93%. Whether to perform radiotherapy for mature teratomas after surgery remains controversial. Sano (6) points out that radiotherapy should be conducted after surgery to suppress further growth of tumor cells. Jakacki (21) suggests that it is advocated to perform radiotherapy to immature teratomas and teratomas with malignant transformation; while mature teratomas are not typically responsive to radiation therapy, surgery is the only proven treatment modality. Therefore, the clinical experience from physicians really matters in the postoperative treatment choices for patients with mature teratomas.

## Conclusion

MCTs in the sellar region are extremely rare, and their imaging usually lacks specificity. Therefore, it is important to obtain a thorough histological diagnosis. MCTs are benign, and complete surgical excision is the first-line treatment. In selected cases, radiation therapy was conducted in some cases but is not recommended as routine treatment. Whether to perform radiotherapy depends on the physician as there is a lack of evidence on this aspect. Close follow-up is indispensable for patients with MCTs.

## Data Availability Statement

The raw data supporting the conclusions of this article will be made available by the authors, without undue reservation.

## Ethics Statement

Written informed consent was obtained from the participant for the publication of any potentially identifiable images or data included in this article. The authors are accountable for all aspects of the work in ensuring that questions related to the accuracy or integrity of any part of the work are appropriately investigated and resolved.

## Author Contributions

SJ drafted the manuscript. SJ and ZW analyzed the data. YY made the pathological diagnosis and drafted the article of pathological findings. All authors contributed to the article and approved the submitted version.

## Funding

This work was supported by the National Key Research and Development Program of China (2018YFA0108600), the Natural Science Foundation of Beijing Municipality (7182134), the CAMS Initiative for Innovative Medicine (2016-I2M-1-017), Beijing Nova Program (Z181100006218003), and the National Natural Science Foundation of China (82170799).

## Conflict of Interest

The authors declare that the research was conducted in the absence of any commercial or financial relationships that could be construed as a potential conflict of interest.

## Publisher’s Note

All claims expressed in this article are solely those of the authors and do not necessarily represent those of their affiliated organizations, or those of the publisher, the editors and the reviewers. Any product that may be evaluated in this article, or claim that may be made by its manufacturer, is not guaranteed or endorsed by the publisher.
